# Novel amidases of two *Aminobacter* sp. strains: Biotransformation experiments and elucidation of gene sequences

**DOI:** 10.1186/2191-0855-2-33

**Published:** 2012-06-27

**Authors:** Ulrike Engel, Christoph Syldatk, Jens Rudat

**Affiliations:** 1 Karlsruhe Institute of Technology (KIT), Institute of Process Engineering in Life Sciences: Section II: Technical Biology, Engler-Bunte-Ring 1, 76131, Karlsruhe, Germany

**Keywords:** Beta-amino acid, Dihydropyrimidinase, Hydantoinase, Carbamoylase

## Abstract

The amidase activities of two *Aminobacter* sp. strains (DSM24754 and DSM24755) towards the aryl-substituted substrates phenylhydantoin, indolylmethyl hydantoin, D,L-6-phenyl-5,6-dihydrouracil (PheDU) and *para*-chloro-D,L-6-phenyl-5,6-dihydrouracil were compared. Both strains showed hydantoinase and dihydropyrimidinase activity by hydrolyzing all substrates to the corresponding *N*-carbamoyl-α- or *N*-carbamoyl-β-amino acids. However, carbamoylase activity and thus a further degradation of these products to α- and β-amino acids was not detected. Additionally, the genes coding for a dihydropyrimidinase and a carbamoylase of *Aminobacter* sp. DSM24754 were elucidated. For *Aminobacter* sp. DSM24755 a dihydropyrimidinase gene flanked by two genes coding for putative ABC transporter proteins was detected. The deduced amino acid sequences of both dihydropyrimidinases are highly similar to the well-studied dihydropyrimidinase of *Sinorhizobium meliloti* CECT4114. The latter enzyme is reported to accept substituted hydantoins and dihydropyrimidines as substrates. The deduced amino acid sequence of the carbamoylase gene shows a high similarity to the very thermostable enzyme of *Pseudomonas* sp. KNK003A.

## Introduction

Hydantoinases (EC 3.5.2.2) were thought to be the microbial counterparts of eukaryotic dihydropyrimidinases. For this reason the terms hydantoinase and dihydropyrimidinase are used synonymously in EC nomenclature. The eukaryotic enzymes catalyze the second step in the reductive pyrimidine degradation pathway by hydrolyzing the dihydropyrimidines dihydrouracil and dihydrothymine to the corresponding *N*-carbamoyl-β-amino acids (Vogels and van der Drift 1976). However, several hydantoinases are reported to lack the ability of hydrolyzing these natural substrates, e.g. D-hydantoinase from *Bacillus thermocatenulatus* GH-2, phenylhydantoinase from *Escherichia coli* and hydantoinase from *Agrobacterium* sp. IP 1–671 (Kim et al. 2000; Park et al. 1999;Runser and Meyer 1993). Therefore the natural function of hydantoinases is still unclear.

**Figure 1:**
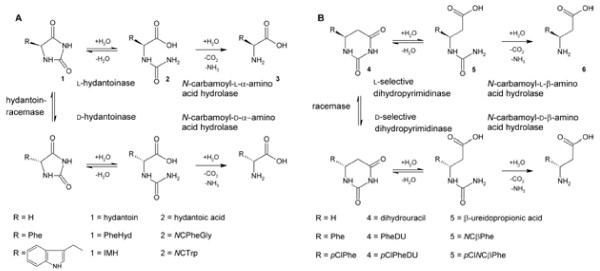
**A) Hydantoinase process for the synthesis of chiral D- or L-α-amino acids starting from racemic 5’-monosubstituted hydantoins via *N*-carbamoyl-α-amino acids applying a hydantoin racemase, a specific hydantoinase and a specific *N*-carbamoyl-α-amino acid hydrolase; B) proposed modified hydantoinase process for the synthesis of chiral D- or L-β-amino acids starting from racemic 6’-monosubstituted dihydropyrimidines via *N*-carbamoyl-β-amino acids applying a racemase, a dihydropyrimidinase and a *N*-carbamoyl-β-amino acid hydrolase; (1) 5’-monosubstituted hydantoin, (2) *N*-carbamoyl-α-amino acid, (3) α-amino acid, (4) 6’-monosubstituted dihydropyrimidine, (5) *N*-carbamoyl-β-amino acid, (6) β-amino acid; PheHyd: phenylhydantoin, *N*CPheGly: *N*-carbamoyl-α-phenylglycine, PheDU: phenyldihydrouracil, *N*CβPhe: *N*-carbamoyl-β-phenylalanine, IMH: indolymethyl hydantoin, *N*CTrp: *N*-carbamoyl-α-tryptophan, *p*ClPheDU: *para*-chloro-phenyldihydrouracil, *p*Cl*N*CβPhe: *para*-chloro-*N*-carbamoyl-β-phenylalanine.**

Apart from that, hydantoinases are of high interest as they are utilized for the biocatalytic production of unnatural enantiopure α-amino acids. In the so called hydantoinase process a racemic hydantoin is converted to a D- or L-*N*-carbamoyl-α-amino acid and subsequently to a chiral D- or L-α-amino acid applying a hydantoin racemase, a D- or L-specific hydantoinase and finally a D- or L-specific *N*-carbamoylase (see Figure 1A). This industrially applied process has a theoretical yield of 100%. Nowadays there is also a rising demand for optically pure β-amino acids. These compounds are promising building blocks for pharmaceuticals and fine chemicals (Seebach and Gardiner 2008). However, the efficient production of chiral β-amino acids is still a challenging task (Weiner et al. 2010).

In a previous study we tested the potential of using a modified hydantoinase process for the production of optically pure β-amino acids (see Figure 1B). We demonstrated that three recombinant D-hydantoinases were able to convert aryl-substituted dihydropyrimidines to the corresponding *N*-carbamoyl-β-amino acids. Additionally, we detected several bacterial strains exhibiting activity towards aryl-substituted dihydropyrimidines. Several bacterial isolates, among them *Aminobacter* sp. DSM24755, showed an enantioselective conversion of phenyldihydrouracil (Engel et al. 2011).

Here we report the dihydropyrimidinase and hydantoinase activities of two *Aminobacter* strains. Moreover the genes coding for a carbamoylase and a dihydropyrimidinase for *Aminobacter* sp. DSM24754 and a gene coding for another dihydropyrimidinase for *Aminobacter* sp. DSM24755 are described.

## Materials and methods

### Chemicals

Chemicals were of reagent grade and obtained from commercial sources if not stated otherwise. D,L-5-Indolylmethyl-*N*-3-methyl hydantoin (CH_3_-IMH) was supplied by former Degussa AG (now Evonik Industries AG). D-β-Phenylalanine (D-βPhe) and L-β-phenylalanine (L-βPhe) were obtained from Pep-Tech Corporation (Burlington, USA).

*para*-Chloro-D,L-phenyldihydrouracil (*p*ClPheDU) was provided from Fraunhofer Institute for Chemical Technology (Pfinztal Germany). Phenylhydantoin (PheHyd), 5-indolylmethyl hydantoin (IMH) and *N*-carbamoyl-α-tryptophan (*N*CTrp) were synthesized according to Stark and Smyth 1963; and Suzuki et al. 1973. Phenyldihydrouracil (PheDU), *N-rac*-carbamoyl-β-phenylalanine (*N*CβPhe) and L-*N*CβPhe as standard for HPLC analysis were prepared according to Dakin and Dudley 1914 and Dürr 2007. The purity was proven by HPLC and ^1^ H-NMR. PheDU used for biotransformation experiments and *para*-chloro-D,L-*N*-carbamoyl-β-phenylalanine (*p*ClNCβPhe) were prepared as described elsewhere (Engel et al. 2011).

### Media

The following media were used: Lysogeny broth (LB) medium modified after Bertani 1951 was used for cultivation of strains for biotransformation assays and growing cells for DNA extraction: 10 g/L Bacto-tryptone, 5 g/L yeast extract and 5 g/L NaCl. The pH was adjusted to 7.2 with NaOH. For LBi medium, 0.1 g/L CH_3_-IMH was added to LB.

### Bacterial strains

The strains *Aminobacter* sp. DSM24754 and *Aminobacter* sp. DSM24755 were kindly provided by Dr. A. Puñal and first described by Engel et al. 2011.

### Cultivation conditions

A bacterial colony was inoculated in 4 mL LB at 30°C with 140 rpm overnight. The resulting preculture was added to 100 mL LB in a 1 L shaking flask and incubated at 30°C and 140 rpm.

### Assay of enzyme activity

Cells were harvested in the late exponential growth phase by centrifugation (8000 x g, 10 min, 12°C). The supernatant was discarded and the cells were washed twice with potassium phosphate buffer (0.1 M, pH 8) followed by centrifugation. Finally resting cells were obtained by resuspending the cells in a small volume of the same buffer.

Preparation of substrate solutions: The assay substrates PheDU, PheHyd and IMH were dissolved in potassium phosphate buffer (0.1 M, pH 8) to a concentration of 4 mM assisted by heating to 70°C for 30 min. *p*ClPheDU was dissolved in DMSO to a concentration of 400 mM due to its poor water solubility. *N*CβPhe and *N*CTrp were dissolved to a concentration of 4 mM in potassium phosphate buffer (0.1 M, pH 8).

Biotransformation reactions with PheDU, IMH, PheHyd, *N*CTrp or *N*CβPhe were started by the addition of 500 μL substrate solution to 500 μL suspension of resting cells. For biotransformation reactions with *p*ClPheDU 5 μL of this substrate solution were added to 495 μL of potassium phosphate buffer (0.1 M, pH 8) and subsequently biocatalysis was started by adding 500 μL suspension of resting cells. All assays were carried out in a thermomixer at 40°C, 1400 rpm for 24 h. Reactions were stopped by centrifugation (13.000 x g, 1 min). Supernatants were harvested and stored at −28°C until analysis.

### Analysis

All substrate and product concentrations were analyzed by HPLC on an Agilent 1100 system (Agilent Technologies, Santa Clara, USA) using a Nucleodur 100–5 C18 ec column (Macherey-Nagel, Germany). The mobile phase for the analysis of PheDU, *N*CβPhe, βPhe, IMH, *N*CTrp, tryptophan (Trp), PheHyd, *N*-carbamoyl-α-phenylglycine (*N*CPheGly) and phenylglycine (PheGly) consisted of 20% MeOH/80% (0.1% (v/v) H_3_PO_4_ pH 3.0 (NaOH)). The flow rate was 0.8 mL/min, the temperature 30°C and the detection wavelength 210 nm. For analysis of *p*ClPheDU and *p*Cl*N*CβPhe the mobile phase was composed of 40% MeOH/60% 0.04 M potassium phosphate buffer pH 6.5. The flow rate was 0.8 min, the temperature 30°C and the detection wavelength 210 nm.

### DNA preparation and sequencing of amidase genes

To extract genomic DNA from bacterial isolates the ZR Soil Microbe DNA Kit™ was applied according to the manufacturer’s instructions. Quality and quantity of genomic DNA were controlled by agarose gel electrophoresis using 1% agarose in TBE buffer. The gel was stained with ethidium bromide solution and analyzed under UV light.

Elucidation of amidase gene sequences is based on dihydropyrimidinase gene fragments amplified and provided by Dr. A. Puñal (Karlsruhe Institute of Technology (KIT) former University of Karlsruhe) with the primers dhyd-f (AAACGGTT) (5’-GCCGCAGCATGCGGNGGNACNAC-3’) and dhyd-r (DADIVIWDPNGE) (5’-CACCATTAGGGTCCCATATGACTADRTCNGCRT-3’) for the strain *Aminobacter* sp. DSM24754 and the degenerate primers dhp-f (AAAFGG) (5’-GCSGCVTTYGGNGGNACNAC-3’) and dhp-r (VHAENG) (5’-TCNCCRTTYTCNGCRTGNAC-3’) for the strain *Aminobacter* sp. DSM24755 (Dürr 2007; Lin et al. 2005). The complete gene sequences were amplified by performing a modified TAIL-PCR. For the first TAIL-PCR the arbitrary degenerate primers AD1 and AD2 according to Liu and Whittier 1995 and the specific primers 728Tf1 (5’-GCTGCATATGTGCGTCAATGGCTGG-3’) and 728Tf2 (5’-GAGCGTGGCGTCAACACCTTCAAG-3’) for strain *Aminobacter* sp. DSM24754 and the specific primers 735Tf1 (5’-GTCGACAAGGGCATCACCTCGTTC-3’) and 735Tf2 (5’-GGTGGACGACGACGAGATGTATTCG-3’) for strain *Aminobacter* sp. DSM24755, synthesized by MWG-Biotech (Germany), were used. In contrast to the originally described method PCR products were separated after the secondary PCR on a 1% agarose gel. PCR products were purified with MinElute Gel Extraction Kit (Qiagen, Hilden Germany), ligated into pDrive PCR cloning vector (Qiagen, Hilden Germany) and sequenced (GATC Biotech AG, Germany). Subsequently the sequences were assembled with the known fragments. With the newly identified sequences further specific primers were designed and TAIL-PCR was conducted as described above until the gene sequences were elucidated completely. Sequence fragments were aligned using BioEdit program, BLAST was applied for comparison with other sequences. ClustalW2 was used for global sequence alignments to compare the new *Aminobacter* sequences with sequences in the NCBI database (Altschul et al. 1997; Altschul et al. 1990; Chenna et al. 2003). The aligned sequences were printed with ESPript (Gouet et al. 1999). For comparison of the *Aminobacter* sp. DSM24754 gene cluster with other prokaryotic genomes the program Comparative Genome Cluster Viewer (CGCV, Revanna et al. 2009) was applied.

## Results

### Biotransformation experiments

**Figure 2:**
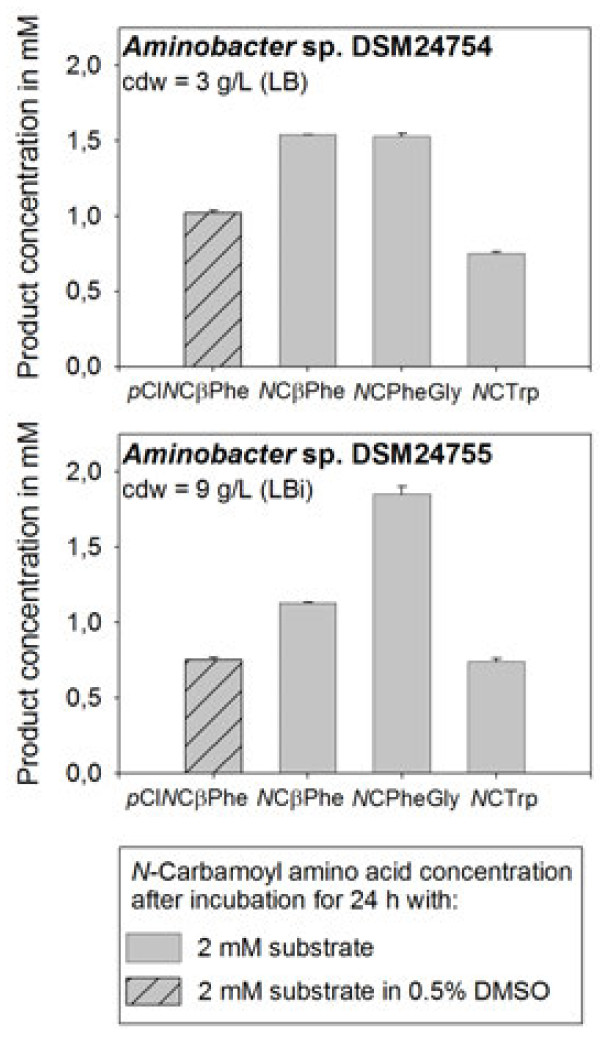
**Product concentrations measured after 24 h of resting cell biotransformation experiments with *Aminobacter *sp. DSM24754 compared to the results determined for *Aminobacter sp*. DSM24755 with four different substrates: *para***-**Chloro-phenyldihydrouracil (pClPheDU) is converted to *para*-chloro-*N*-carbamoyl-β-phenylalanine** (***p*ClNCβPhe)**, **phenyldihydrouracil (PheDU) is converted to *N***-**carbamoyl-β-phenylalanine (*N*CβPhe); phenylhydantoin (PheHyd) is converted to *N*-carbamoyl-α-phenylglycine (*N*CPheGly) and indolymethyl hydantoin (IMH) is converted to *N*-carbamoyl-α-tryptophan** (***N*CTrp).** The substrate concentrations were 2 mM; cdw = cell dry weight.

Due to previous results *Aminobacter* sp.DSM24754 was cultivated in LB and strain *Aminobacter* sp. DSM24755 in LBi for all experiments (Engel et al. 2011). The dihydropyrimidinase, hydantoinase and carbamoylase activities of *Aminobacter* sp. DSM24754 were tested. The results are summarized in Figure 2. Besides dihydropyrimidinase activity towards PheDU and *p*ClPheDU hydantoinase activity towards PheHyd and IMH was detected for both strains. Under the chosen conditions PheDU and PheHyd were the best substrates for *Aminobacter* sp. DSM24754 and PheHyd was the best substrate for *Aminobacter* sp. DSM24755. None of the *Aminobacter* strains exhibited carbamoylase activity towards the *N*-carbamoyl-β-amino acid *N*CβPhe or the *N*-carbamoyl-α-amino acid *N*CTrp.

### Identification of amidase genes

A modified TAIL-PCR was conducted for both strains based on dihydropyrimidinase gene fragments. With this method a 3252 base pairs (bp) long DNA fragment from strain *Aminobacter* sp. DSM24754 and a 3127 bp long DNA fragment from *Aminobacter* sp. DSM24755 were amplified and subsequently sequenced.

#### Sequence analysis of the genomic DNA fragment from Aminobacter sp. DSM24754

The 3252 bp genomic DNA fragment of *Aminobacter* sp. DSM24754 has an overall GC content of 62% and comprises two complete open reading frames (ORFs) and one incomplete ORF all starting with an ATG start codon (see Table 1).

**Table 1:** **Location and properties of genes and deduced proteins of the sequenced fragments of *Aminobacter *sp. DSM24754 and *Aminobacter *sp. DSM24755 respectively**

***Aminobacter *sp.**	**Gene**	**Nucleotide position**	**GC (%)**	**Putative RBS**	**Protein length (aa)**	**Calculated molecular mass (kDa)**	**Protein with highest identity in BLAST**	**Accession number**	**Organism**	**Identical amino acids (%)**
DSM24754	ORF1	274-1215	62	TGAGGAGGTAAGACC**ATG**	313	35	*N*-carbamoyl-D-amino acid amidohydrolase	BAD00008.1	*Pseudomonas* sp. KNK003A	85
	ORF2	(1238)/	(62.5)/	*ATG*ACGGCAATCAATGGGAGAACGAGC**ATG**	(494)/	(53.8)/	putative dihydropyrimidinase	YP_675206	*Mesorhizobium* sp. BNC1	83
		1265-2722	62.7		485	52.9				
	ORF3	3252-2818	59.8	-	-	-	hypothetical protein	YP_002542208	*Agrobacterium radiobacter* K84	78
DSM24755	ORF1	80-1531	63.6	TAGGGGAACTGAAGAA**ATG**	483	53.2	putative phenylhydantoinase	NP_103173.1	*Mesorhizobium loti* MAFF303099	90
	ORF2	1780-2601	62.8	TGGGGTAAAACCGGGCA**ATG**	273	67.5	putative ATP-binding protein of ABC transporter	NP_103169.1	*Mesorhizobium loti* MAFF303099	91
	ORF3	2607-3127	62.8	-	-	-	putative permease protein of ABC transporter	ZP_09089625.1	*Mesorhizobium amorphae* CCNWGS012*3*	88

(−) no data available, due to an incomplete open reading frame. GC = guanine-cytosine content; aa = amino acids, (*) = values in brackets are for the 1485 bp long gene fragment of ORF2; underlined sequence parts = putative Shine-Dalgarno sequence (RBS), sequence in boldface = assumed start codon, sequence in italics = potential alternative start codon.

The first ORF consists of 942 bp coding for a protein of 313 amino acids. It has the highest amino acid identity of 85% to the primary sequence of non-putative *N*-carbamoyl-D-amino acid amidohydrolase of *Pseudomonas* sp. KNK003A (BAD00008.1). ORF2 is located downstream to ORF1 and points into the same direction. It is either 1485 bp or 1458 bp long, as there is a second ATG start codon 27 bp downstream to the first. The real start codon has to be determined by a heterologous expression of the longer and the shorter version of the gene and subsequent activity tests. However, most of the similar enzymes in the database are aligning beginning with the second start codon. A possible Shine-Dalgarno sequence (RBS) is only detected upstream the second ATG codon (see Table 1). For these reasons all following identity data are related to this shorter gene product coding for a protein of 485 amino acids. This protein shares highest amino acid identity (83%) with a putative dihydropyrimidinase of *Mesorhizobium* sp. BNC1 (YP_675206). The third ORF is 326 bp long, incomplete and points into the opposite direction. Its deduced amino acid sequence shows highest identity of 78% to a hypothetical protein of *Agrobacterium radiobacter* K84 (YP_002542208.1). The possible function of the gene product of ORF3 remains unclear as it exhibits no clear similarity to non-putative proteins.

The sequence of the 3252 bp genomic DNA fragment of *Aminobacter* sp. DSM24754 was deposited at EMBL database [HE651322].

#### Sequence analysis of the genomic DNA fragment from Aminobacter sp. DSM24755

The 3127 bp DNA fragment has an overall GC content of 63% and harbors two complete ORFs and one incomplete ORF (see Table 1). All ORFs point into the same direction and start with an ATG start codon.

The first ORF is 1452 bp long and encodes a protein of 483 amino acids. This protein shows highest amino acid identity (90%) to the putative dihydropyrimidinase of *Mesorhizobium loti* MAFF303099 (NP_103173.1). A second ORF of 822 bp is located downstream to ORF1. Its deduced amino acid sequence displays highest identity (91%) to a putative ATP-binding protein of an ABC transporter of *Mesorhizobium loti* MAFF303099 (NP_103169.1). The incomplete third ORF starts only 4 bp downstream to ORF2 and consists of 521 bp. The deduced partial amino acid sequence exhibits highest identity (88%) to the transmembrane component of an ABC transporter of *Mesorhizobium amorphae* CCNWGS0123 (ZP_09089625.1).

The 3127 bp genomic DNA fragment of *Aminobacter* sp. DSM24755 was deposited at EMBL database [HE651323].

The two genes coding for putative dihydropyrimidinases of the *Aminobacter* sp. strains DSM24754 and DSM24755 have an overall gene sequence identity of 74%. The identity of the deduced amino acid sequences is 68%.

## Discussion

### Biotransformation results

In this study the dihydropyrimidine, hydantoin and carbamoyl amino acid hydrolyzing abilities of *Aminobacter* sp. DSM24754 were determined. Both *Aminobacter* strains showed dihydropyrimidinase and hydantoinase activity towards PheDU, *p*ClPheDU, PheHyd and IMH by converting these substrates to the corresponding *N*-carbamoyl-β- and *N*-carbamoyl-α-amino acids. *Aminobacter* sp. DSM24754 hydrolyzed the same amount of PheHyd and PheDU under the chosen conditions. *Aminobacter* sp. DSM24755 also showed a high conversion of PheHyd but in contrast to the results obtained for the other *Aminobacter* strain the dihydropyrimidine PheDU was not hydrolyzed to the same extend. This may be due to the fact that a D-selectivity for PheDU is described for *Aminobacter* sp. DSM24755 while *Aminobacter* sp. DSM24754 is reported to be unselective for this substrate (Engel et al. 2011). Nothing is known about the stereoselectivity for PheHyd of both biocatalysts. But this substrate is known to spontaneously racemize under alkaline conditions because of its keto-enol-tautomerism (Pietzsch and Syldatk 1995). For this reason a potential stereoselectivity of the biocatalyst would probably not influence the biotransformation reaction. The dihydropyrimidine PheDU cannot racemize spontaneously due to its different chemical structure. Thus a stereoselective biocatalyst would hydrolyze this substrate less effective.

When only the hydantoins PheHyd and IMH were compared PheHyd appeared to be the better substrate for both biocatalysts as a higher amount of this compound was degraded, respectively. This hydantoin is composed of a phenyl group directly bound to the hydantoin ring, which is highly similar to PheDU consisting of a phenyl group linked to a dihydropyrimidine ring. By contrast, the bulky aromatic indol ring of IMH is connected to the hydantoin ring via a methyl bridge. This difference in substrate structure may be a reason for the differences observed in our experiments. In general D-hydantoinases preferably hydrolyze phenyl-substituted hydantoins while L-hydantoinases show higher activities towards benzyl-substituted hydantoins such as IMH which is assumed to be a consequence of their different three-dimensional structures (Abendroth et al. 2002a).

A further degradation of the resulting *N*-carbamoyl amino acids was not observed. Additionally, no carbamoylase activities were detectable in resting cell biotransformation experiments directly applying *N*CTrp and *N*CβPhe as substrates. We see two possible explanations for these results: Either (i) there are no genes coding for carbamoylases present in the genomes of the two tested *Aminobacter* strains or (ii) the experimental settings prevented the detection of carbamoylase activity. For example the expression of carbamoylases is reported to be inducible (Mei et al. 2008). Consequently the lack of an inducer during growth of *Aminobacter* sp. DSM24754 or the use of the wrong inducer (CH_3_-IMH) during growth of *Aminobacter* sp. DSM24755 may account for not detectable carbamoylase activities. Otherwise it may be that carbamoylases were expressed but not active with the substrates tested. Explanation (ii) seems to be more plausible as at least for strain *Aminobacter* sp. DSM24754 a gene product coding for a D-carbamoylase was detected (see above).

### Comparison with other functionally related carbamoylases

**Figure 3:**
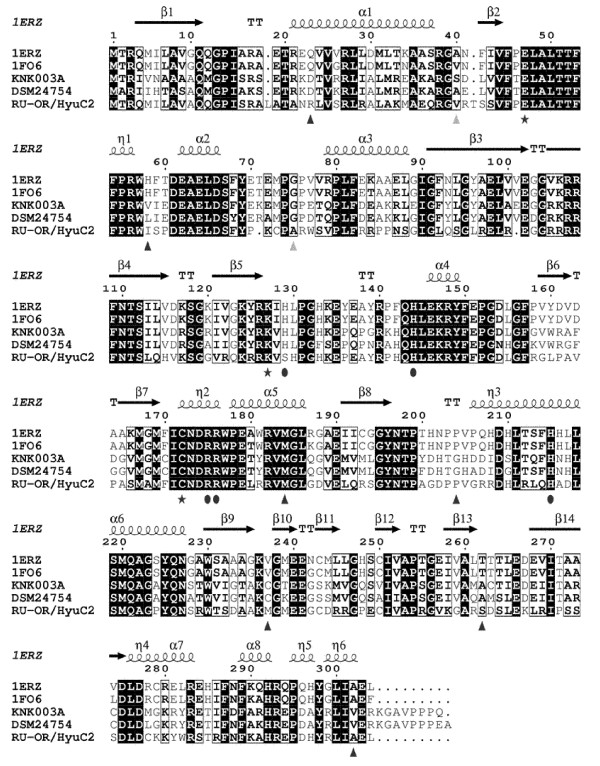
**Alignment of primary amino acid sequences of the non-putative D-carbamoylases of *Agrobacterium tumefaciens *RU-OR (HyuC2; ABS11194.1), *Pseudomonas *sp. KNK003A (BAD00008.1), *Agrobacterium radiobacter *NRRL B11291 (1FO6), *Agrobacterium *sp. KNK712 (1ERZ) and the deduced carbamoylase sequence of *Aminobacter *sp.DSM24754.** The dark shading symbolizes identical residues while bold letters represents similar residues. (⋆) residues identified as catalytic triad, () residues assumed to play a key role in substrate recognition (Chen et al. 2003; Wang et al. 2001; Nakai et al. 2000), (▴) residues supposed to influence thermal stability (Oh et al. 2002; Ikenaka et al. 1999; Chiu et al. 2006; Chiang et al. 2008), (▴) residues potentially influencing oxidative stability (Oh et al. 2002).

For strain *Aminobacter* sp. DSM24754 a gene coding for a carbamoylase was identified and the deduced amino acid sequence showed highest amino acid identity (85%) to the D-carbamoylase of *Pseudomonas* sp. KNK003A (BAD00008.1, Ikenaka et al. 1998). Furthermore it exhibited 49–58% identity to three well studied D-carbamoylases of *Agrobacterium radiobacter* CCRC14924 (1FO6) *Agrobacterium* sp. KNK712 (1ERZ) and *Agrobacterium tumefaciens* RU-OR (HyuC2; ABS11194.1) within a global alignment (see Table 2 and Figure 3; Wang et al. 2001; Nakai et al. 2000; Jiwaji et al. 2009).

**Table 2:** **Comparison of the primary amino acid sequence of the putative carbamoylase found in *Aminobacter *sp**

**Amino acid sequence identities and similarities of carbamoylases**		**DSM24754**	**BAD00008.1**	**1FO6**	**1ERZ**	**ABS11194.1**
		**Identity %**				
*Aminobacter* sp.			85	58	57	49
**DSM24754**						
*Pseudomonas* sp.		92		59	58	51
KNK003 **BAD00008.1**						
*Agrobacterium radiobacter*		68	70		96	57
CCRC14924 **1FO6**						
*A.* sp. KNK712 **1ERZ**		69	70	97		56
*A. tumefaciens* RU-OR		56	57	64	93	
HyuC2 **ABS11194.1**						

Comparison of the primary amino acid sequence of the putative carbamoylase found in *Aminobacter sp*. DSM24754 with the amino acid sequences of *Pseudomonas sp*. KNK003 (BAD00008.1), *Agrobacterium radiobacter* CCRC14924 (1FO6), *Agrobacterium sp*. KNK712 (1ERZ) and *Agrobacterium tumefaciens* RU-OR HyuC2 (ABS11194.1) within a global alignment.

In this alignment important residues were annotated and the residue numbers refer to the protein sequence of *Aminobacter* sp. DSM24754. All of the aligned non-putative enzymes are D-carbamoylases able to catalyze the hydrolytic cleavage of *N*-carbamoyl-α-amino acids. For none of these enzymes activity towards *N*-carbamoyl-β-amino acids is described (Martinez-Rodriguez et al. 2010a). The residues identified as the catalytic triad (Glu47, Lys127, Cys172) and the residues assumed to play a key role in substrate recognition (His129, His144, Arg175, Arg176, His215) are highly conserved for all sequences aligned (Nakai et al. 2000; Wang et al. 2001; Chen et al. 2003). Due to its sequence characteristics the A*minobacter* sp. DSM24754 enzyme can also be classified as D-carbamoylase.

The putative carbamoylase of *Aminobacter* sp. DSM24754 exhibited highest identity to the *Pseudomonas* sp. KNK003A D-carbamoylase, which is described to be the most thermostable carbamoylase known today (Martinez-Rodriguez et al. 2010a). However, the low stability of D-carbamoylases is one major drawback for their efficient use in an industrial hydantoinase process. Therefore various attempts have been made to engineer their thermal and oxidative stability. For the carbamoylase of *A. radiobacter* CCRC14924 it was reported that the residues Gln23, His58, Met184, Val237 and Thr262 influence the stability against temperature and oxidation, the residues Val40 and Gly75 affect the oxidative stability solely (Oh et al. 2002; Chiang et al. 2008), and that the residue Ala302 influences thermostability and catalytic activity (Chiu et al. 2006). Thermal and pH stability of the carbamoylase of *Agrobacterium* sp. KNK712 was shown to be affected by the residues His58, Pro204 and Val237 (Ikenaka et al. 1999). Except for Met184, all residues described to be involved in thermal stability of the two *Agrobacterium* enzymes differ from the residues in the carbamoylase sequences of *Aminobacter* sp. DSM24754 and *Pseudomonas* sp. KNK003A. Remarkably 5 of these 6 substitutions (Asp23, Gly204, Cys237, Ala262, Val302) are identical while one is similar (Val/Leu58) for the both latter enzymes. Furthermore in the D-carbamoylase of *A. radiobacter* CCRC14924 a single mutation of Thr262 to Ala led to a significant increase in oxidative and thermal stability (Oh et al. 2002). The enzymes of *Pseudomonas* sp. KNK003A and *Aminobacter* sp. DSM24754 already possess an Ala residue in this position. Due to its sequences characteristics the *Aminobacter* sp. DSM24754 carbamoylase could have a similar high temperature stability like the *Pseudomonas* sp. KNK003A D-carbamoylase. This is to be studied in more detail within further experiments by its recombinant expression and biochemical characterization.

The recently discovered second carbamoylase of *A. tumefaciens* RU-OR (HyuC2) has as well substitutions in four positions (Arg24, Ile60, Met237, Ser262) compared to the above mentioned seven residues probably important for thermal stability (Jiwaji et al. 2009). Thus the authors assumed that the thermal and oxidative stability of the enzyme may differ from those of the other well characterized *Agrobacterium* enzymes. However, the exchanged residues are neither identical with the residues of *Aminobacter* sp. DSM24754 carbamoylase nor with the corresponding residues of the *Pseudomonas* sp. KNK003A carbamoylase protein sequence.

### Comparison with other functionally related hydantoinases

**Figure 4:**
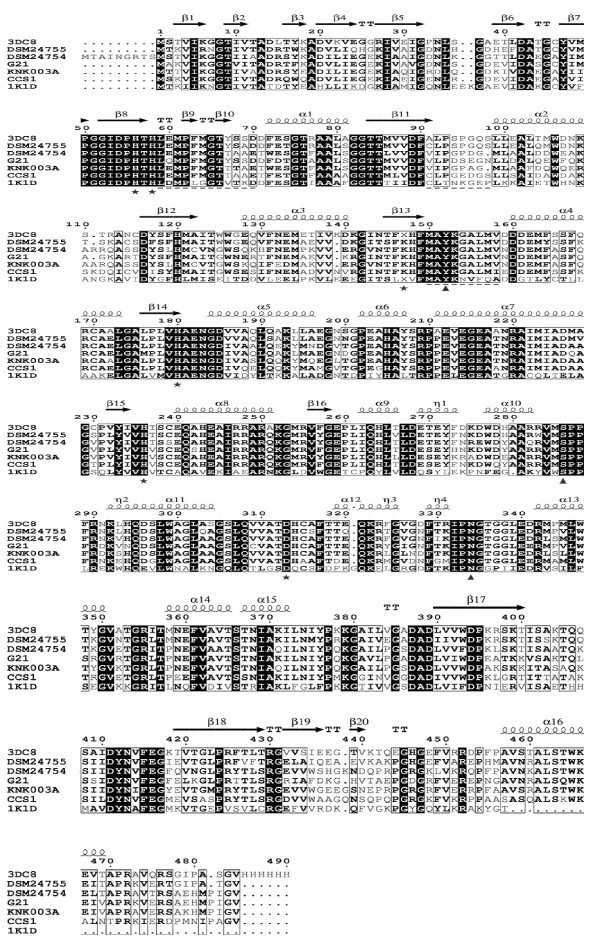
**Alignment of primary amino acid sequences of the non-putative dihydropyrimidinase of *Sinorhizobium meliloti *CECT4114 (3 DC8), D-hydantoinase of *Ochrobactrum *sp. G21 (ABS84244.1), D-hydantoinase of *Pseudomonas *sp. KNK003A (BAE20330.1), *Jannaschia *sp. CCS1 (YP_510647.1) and D-hydantoinase of *Bacillus stearothermophilus *SD1 (1K1D) and the deduced dihydropyrimidinase sequences of *Aminobacter *sp. DSM24754 and *Aminobacter *sp. DSM24755.** The dark shading symbolizes identical residues while bold letters represents similar residues. (- -) stereochemistry gate loops (SGL1: 59–70; SGL2: 91–98, SGL3: 150–158), (⋆) residues assumed to build the catalytic core, (▴) residues assumed to be involved in the recognition the hydantoin ring (Martinez-Rodriguez et al. 2010b).

Within a BLAST search against the protein database the deduced amino acid sequence of DSM24754 ORF2 exhibited highest identity (83%) to a putative dihydropyrimidinase of *Mesorhizobium* sp. BNC1. Surprisingly the second BLAST hit was with the non-putative hydantoinase of *Pseudomonas* sp. KNK003A (BAE20330.1) having an overall identity of 82%. This is the same *Pseudomonas* strain whose carbamoylase showed highest identity to *Aminobacter* sp. DSM24754 carbamoylase (see section above). When compared to other non-putative enzymes (see Table 3 and Figure 4) the identities were 70–73% to the *Ochrobactrum* sp. G21 D-hydantoinase (ABS84244.1), to the D-hydantoinase of *Jannaschia* sp. CCS1 (YP_510647.1) and to the dihydropyrimidinase of *Sinorhizobium meliloti* CECT4114 (3 DC8) (Dürr et al. 2008; Cai et al. 2009; Martinez-Rodriguez et al. 2010b). The identity to the well-studied D-hydantoinase of *Bacillus stearothermophilus* SD1 (1K1D) was 42% (Cheon et al. 2002). The overall identity to the deduced dihydropyrimidinase of *Aminobacter* sp. DSM24755 was 68% only, although both gene products were detected in strains belonging to the same genus.

**Table 3:** **Comparison of the primary amino acid sequences of the putative hydantoinases detected in *Aminobacter *sp**

**Amino acid sequence identities and similarities of hydantoinases**		**DSM24754**	**DSM24755**	**BAE20330.1**	**ABS84244.1**	**YP_510647.1**	**3 DC8**	**K1D**
		**Identity %**						
*Aminobacter* sp. **DSM24754**			69	82	73	73	70	42
*Aminobacter* sp. **DSM24755**		83		68	74	68	79	46
*Pseudomonas* sp. KNK003A **BAE20330.1**		91	82		73	71	69	42
*Ochrobactrum* sp. G21 **ABS84244.1**		86	87	86		69	71	42
*Jannaschia* sp. CCS1 **YP_510647.1**		85	80	83	81		68	42
*Sinorhizobium meliloti* CECT4114 **3 DC8**		81	89	81	84	78		44
*Bacillus stearothermophilus* SD1 **1K1D**		59	64	61	60	59	61	

Comparison of the primary amino acid sequences of the putative hydantoinases detected in *Aminobacter sp*. DSM24754 and *Aminobacter sp*. DSM24755 with the amino acid sequences of *Pseudomonas sp*. KNK003A (BAE20330.1), *Ochrobactrum sp*. G21 (ABS84244.1), *Jannaschia sp*. CCS1 (YP_510647.1), *Sinorhizobium meliloti* CECT4114 3DC8 and *Bacillus stearothermophilus* SD1 (1K1D) ) within a global alignment.

The deduced dihydropyrimidinase of DSM24755 ORF1 showed highest identity of 91% to the putative phenylhydantoinase of *Mesorhizobium loti* MAFF303099 in a BLAST search. The highest overall identity of 79% to a non-putative protein was detected with the dihydropyrimidinase of *S. meliloti* CECT4114. A global alignment with further non-putative enzymes (see Table 3 and Figure 4) showed that the amino acid identities to *Ochrobactrum* sp. G21 D-hydantoinase, to the D-hydantoinase of *Pseudomonas* sp. KNK003A and to the D-hydantoinase of *Jannaschia* sp. CCS1 were 74% to 68%. The primary amino acid identity to the D-hydantoinase of the *B. stearothermophilus* SD1 was only 46%.

In the global alignment of the protein sequences the residues described to be important for structure or function were annotated (see Figure 4). The following residue numbers refer to the deduced amino acid sequence of *Aminobacter* sp. DSM24755 dihydropyrimidinase. Like other dihydropyrimidinases the deduced *Aminobacter* sp. DSM24754 and *Aminobacter* sp. DSM24755 enzymes possess the highly conserved GxxDxHxH motif (residues 51–58) (May et al. 1998). Together with the histidin residues of this motif the residues Lys146, His179, His235 and Asp312 are suggested to form the catalytic core and are as well completely conserved within the aligned proteins. Furthermore the lysine residue (Lys146) is post-translationally carboxylated in most known hydantoinases/dihydropyrimidinases (Abendroth et al. 2002b; Cheon et al. 2002). It is confirmed for several hydantoinases/dihydropyrimidinases that these active site residues form a binuclear center with divalent metal ions (Martinez-Rodriguez et al. 2010b; Zhang et al. 2010). Due to the fact that these residues are completely conserved in the protein sequences of *Aminobacter* sp. DSM24754 and *Aminobacter* sp. DSM24755 and due to the overall high sequence similarity with the well-studied dihydropyrimidinase of *S. meliloti* CECT4114 a metal dependence of these newly described enzymes is most likely.

The C-termini of hydantoinases are supposed to be involved in quaternary structure composition (Kim and Kim 1998, 2002). This region was described to be non-homologous among microbial hydantoinases. However, it was recently reported that the C-termini of hydantoinases are highly conserved for *α-Proteobacteria* (Martinez-Rodriguez et al. 2010b). The C-terminal regions of the deduced amino acid sequences of the *α-Proteobacteria* strains *Aminobacter* sp. DSM24754 and *Aminobacter* sp. DSM24755 support this assumption. They are highly homologous to the C-termini of *S. meliloti* CECT4114 dihydropyrimidinase, *Ochrobactrum* sp. G21 d-hydantoinase, and *Jannaschia* sp. CCS1 hydantoinase (see Figure 4).

For *S. meliloti* CECT4114 it is assumed that the substrate’s hydantoin ring is recognized by residues Tyr152, Ser286 and Asn334, which are highly conserved among hydantoinases (Martinez-Rodriguez et al. 2010b). The corresponding residues in *Aminobacter* sp. DSM24754 and *Aminobacter* sp. DSM24755 dihydropyrimidinases are identical. The exocyclic side chain of the substrate is reported to be recognized by the so called stereochemistry gate loops (SGL), which are less conserved. It was suggested that these SGLs may be involved in determining the substrate specificity of these enzymes (Cheon et al. 2002). For the two *Aminobacter* sp. dihydropyrimidinases the residues of SGL3 (see Figure 4) are identical to each other and to *S. meliloti* CECT4114 dihydropyrimidinase, *Ochrobactrum* sp. G21 hydantoinase and *Pseudomonas* sp. KNK003A hydantoinase and are highly similar to the *Jannaschia* sp. CCS1 hydantoinase. There are bigger differences with regard to SGL1 and SGL2 among the two *Aminobacter* enzymes. The *Aminobacter* sp. DSM24754 dihydropyrimidinase SGL1 and SGL2 residues are nearly identical to residues of SGL1 and SGL2 in *Jannaschia* sp. CCS1 hydantoinase. In contrast the SGL1 and SGL2 residues of *Aminobacter* sp. DSM24755 dihydropyrimidinase almost completely match with the residues of *S. meliloti* CECT4114 dihydropyrimidinase. These results could indicate that the substrate specificity of the two *Aminobacter* sp. dihydropyrimidinases may be slightly different. This would correspond to the results obtained in the resting cell biotransformation experiments with the two strains.

In resting cell biocatalysis experiments *Aminobacter* sp. DSM24754 and *Aminobacter* sp. DSM24755 exhibited hydantoinase and dihydropyrimidinase activity. This raises the question whether these dihydropyrimidinase and the hydantoinase activities originate from one enzyme or two different enzymes, a hydantoinase and a dihydropyrimidinase in the respective strain. The *S. meliloti* CECT4114 dihydropyrimidinase is described to hydrolyze substituted five-membered and also six-membered ring substrates (Martinez-Rodriguez et al. 2010b). In a previous study we reported that *Ochrobactrum* sp. G21 hydantoinase, *Delftia* sp. I24 hydantoinase and *Arthrobacter crystallopoietes* DSM20117 hydantoinase can act on substituted hydantoins and on aryl-substituted dihydropyrimidines (Engel et al. 2011). The hydantoinase of *Jannaschia* sp. CCS1 is described to accept hydantoins but showed highest activity towards dihydrouracil (Cai et al. 2009). However, nothing is reported concerning the substrate specificity of *Pseudomonas* sp. KNK003A hydantoinase (Ikenaka et al. 1998). Due to the fact that most of the similar hydantoinases/dihydropyrimidinases have hydantoinase and dihydropyrimidinase activity we hypothesize that the detected deduced dihydropyrimidinases of *Aminobacter* sp. DSM24754 and *Aminobacter* sp. DSM24755 are responsible for both measured activities as well. The major difference was that strain *Aminobacter* sp. DSM24755 showed a D-stereoselectivity for phenyldihydrouracil while strain *Aminobacter* sp. DSM24754 did not (Engel et al. 2011). Whether this is related to the differences in the primary structure of the dihydropyrimidinases has to be elucidated in further experiments with the pure enzymes.

### Comparison to other hydantoin/dihydropyrimidine cleaving gene clusters

Bacterial hydantoin utilizing (*hyu*) genes especially hydantoinases/dihydropyrimidinases and carbamoylases are often organized in gene clusters (Dürr et al. 2008). Compared to other described *hyu* gene clusters the arrangement of the D-carbamoylase (*hyuC*) gene upstream to the D-hydantoinase (*hyuH*) gene in *Aminobacter* sp. DSM24754 appears to be unusual. To our knowledge *Arthrobacter crystallopoietes* DSM20117 is the only bacterial strain with a similar gene organization and approved hydantoinase and carbamoylase activities reported in literature (Werner et al. 2004). The similarity of the two *Aminobacter* sp. dihydropyrimidinases to the D-hydantoinase protein of *A. crystallopoietes* DSM20117 is 57% and the similarity of *Aminobacter* sp. DSM24754 carbamoylase to the D-carbamoylase protein of *A. crystallopoietes* DSM20117 is 68%.

**Figure 5:**
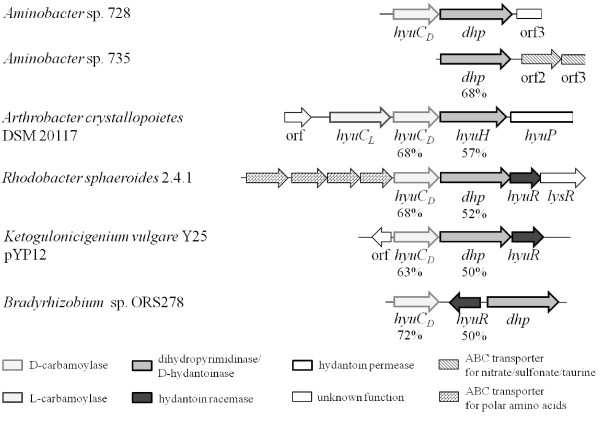
**Comparison of the gene cluster of *Aminobacter *sp.DSM24754 to similar gene clusters.** The gene clusters of *Rhodobacter sphaeroides* 2.4.1 (NC_007494.1), *Ketogulonicigenium vulgare* Y25 pYP12 (NC_014626), *Bradyrhizobium* sp. ORS278 (NC_009445) and the partial sequence of *Arthrobacter crystallopoietes* DSM 20117 (AY185303.1) were obtained from NCBI. The values in % show the similarity of the deduced proteins to the deduced proteins of *Aminobacter* sp. DSM24754.

A database search against prokaryotic genomes resulted in only eight hits showing a comparable gene organization of putative *hyuC* and *hyuH* genes (see Figure 5). In the chromosome of four *Rhodobacter* strains (*R. sphaeroides* ATCC 17025, *R. sphaeroides* ATCC 17029, *R. sphaeroides* 2.4.1, *R. sphaeroides* KD131) and on the plasmid pYP12 of *Ketogulonicigenium vulgare* Y25 the same arrangement of the D-carbamoylase gene and the D-hydantoinase gene as observed in *Aminobacter* sp. DSM24754 was detected. A similar gene organization was found in three *Bradyrhizobium* strains with the difference that a gene coding for a hydantoin racemase pointing in the opposite direction (for *Bradyrhizobium* sp. BTAi1, *Bradyrhizobium* sp. ORS278) or two hypothetical genes (for *B. japonicum* USDA 110) are located between *hyuC* and *hyuH*.

The *Rhodobacter sphaeroides* strains and *Ketogulonicigenium vulgare* Y25 harbor two genes coding for hydantoinases respectively. The second hydantoinase forms a *hyu* gene cluster with a β-ureidopropionase (*R. sphaeroides*) or an L-carbamoylase (*K. vulgare* Y25) located upstream to the hydantoinase gene. The similarity of *Aminobacter* sp. DSM24754 and DSM24755 dihydropyrimidinases to the *R. sphaeroides* and *K. vulgare* Y25 hydantoinases clustering with the β-ureidopropionases or L-carbamoylase is higher (66–89%) than to the hydantoinases clustering with the D-carbamoylases (50–53%).

The *Bradyrhizobium* strains possess three genes coding for D-hydantoinases and harbor three to four genes coding for D-carbamoylases, respectively. However, only one hyu cluster composed of a D-carbamoylase and hydantoinase is found in each strain. The sequence similarity of the *Aminobacter* sp. dihydropyrimidinases to the *Bradyrhizobium* hydantoinases not forming a cluster with a D-carbamoylase is again higher (49–78%) than to the hydantoinases clustering with a D-carbamoylase (47–51%). The similarities of *Bradyrhizobium* D-carbamoylases clustering with a hydantoinase to the *Aminobacter* sp. carbamoylases are 71–74% while the similarities to the other carbamoylases are between 46 and 74%.

This raises the questions whether or not the two *Aminobacter* sp. strains also possess several hydantoinases and carbamoylases and what role the *hyu* genes and their clustering play for these strains.

## Competing interests

The authors declare that they have no competing interests.
